# Supplementary material for: Solar and wind energy enhances drought resilience and groundwater sustainability
Source: Nat Commun. 2019 Nov 6;10:4893. doi: 10.1038/s41467-019-12810-5 (PMC6834588; doi:10.1038/s41467-019-12810-5)
Supplement: Supplementary file 1 — Supplementary Information [file 41467_2019_12810_MOESM1_ESM.pdf]

Supplementary Information for

“Solar and wind energy enhances drought resilience and groundwater sustainability”

He et al.

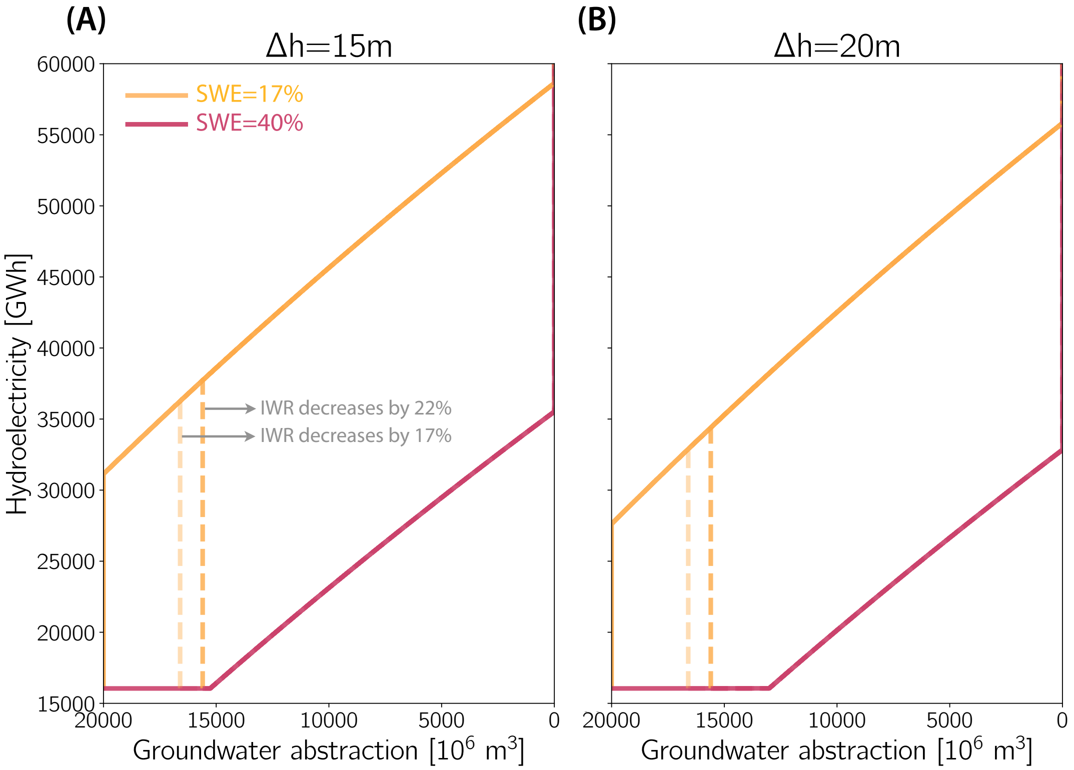

Supplementary Figure 1. Sensitivity tests showing optimal expansion paths (EPs) under current (yellow lines, 17%) and future (red lines, 40%) penetration of SWE considering changes in irrigation efficiency for (a) $\Delta h = 15m$ and (b) $\Delta h = 20m$. Dashed lines show results considering decreased irrigation water requirement (IWR), given that future irrigation efficiency in California is expected to increase by 17-22% in all year types (i.e., normal, dry, wet)[1]. Note: Under 40% penetration of SWE, red dashed lines and red solid lines overlap with each other, which indicate that increased irrigation efficiency does not influence EPs when penetration of SWE is high.

Station: USGS 11074000

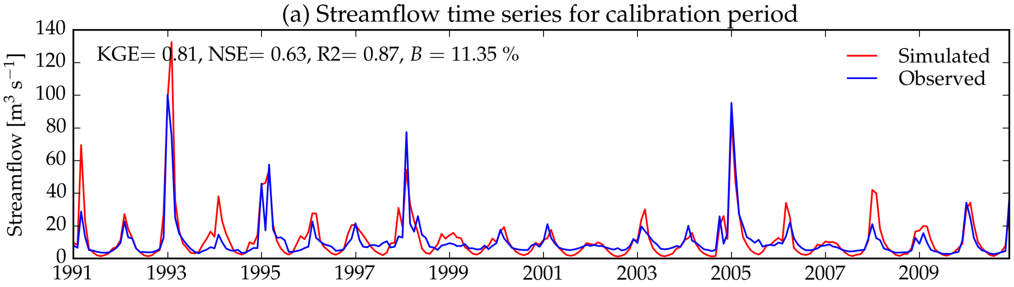

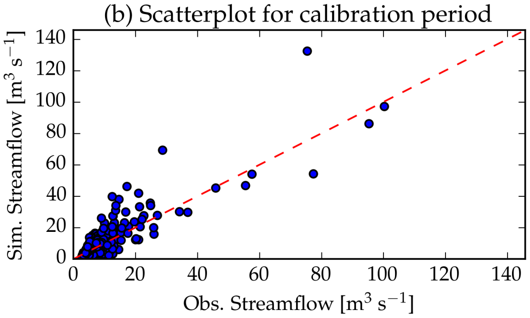

	Obs.	Sim.
KGE		0.806
NS		0.631
NSlog		0.176
R2		0.870
Bias		11.35%
RMSE		8
MAE		4
Mean	11	12
Min	3	1
5 %	4	2
50 %	7	8
95 %	26	36
99 %	77	80
Max	100	133

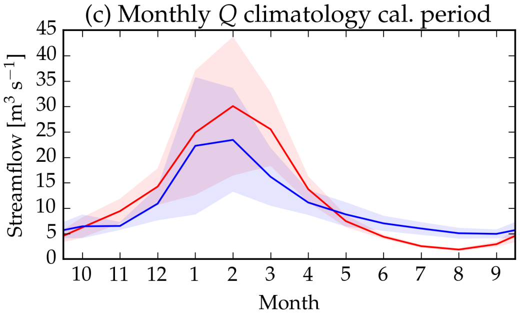

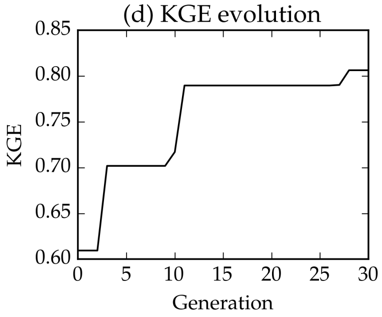

Supplementary Figure 2. Calibration results for USGS gauge 11074000. (a) Observed (blue) and simulated (red) monthly streamflow time series during the calibration period (1991-2010). (b) Scatter plot showing the relationship between monthly observed and simulated streamflow. (c) Monthly climatology of mean observed (blue) and mean simulated (red) streamflow for the water year (from October to September) during the calibration period (1991-2010). Shaded area represents the standard deviation around the mean value for each month. (d) Evolution of Kling-Gupta Efficiency (KGE) metric during the calibration process, which is used as the objective function to be maximized to calibrate the hydrological model. Detailed model performance metrics are summarized in the right table, including KGE, NS (Nash-Sutcliffe coefficient of efficiency) and its log form (NSlog), R^2 (coefficient of determination), Bias, RMSE (root mean squared error), and MAE (mean absolute error). Observed and simulated streamflow statistics are also included in the table, including mean, minimum, maximum, and different quantiles (5%, 50%, 95%, 99%).

Station: USGS 11074000

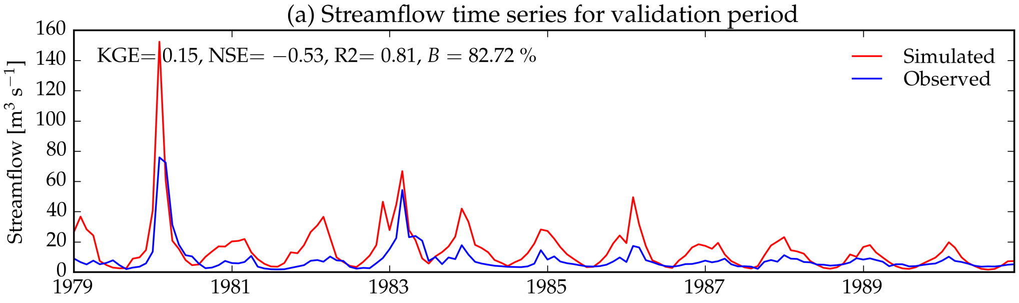

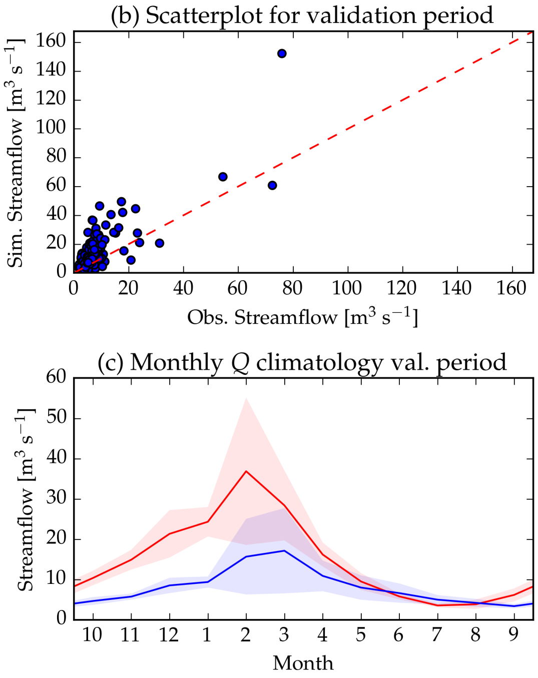

	Obs.	Sim.
KGE		0.146
NS		-0.532
NSlog		-0.558
R2		0.810
Bias		82.72%
RMSE		12
MAE		8
Mean	8	15
Min	2	2
5 %	3	3
50 %	6	11
95 %	20	40
99 %	65	64
Max	76	152

Supplementary Figure 3. Same as Supplementary Figure 2, but for validation.

Station: USGS 11150500

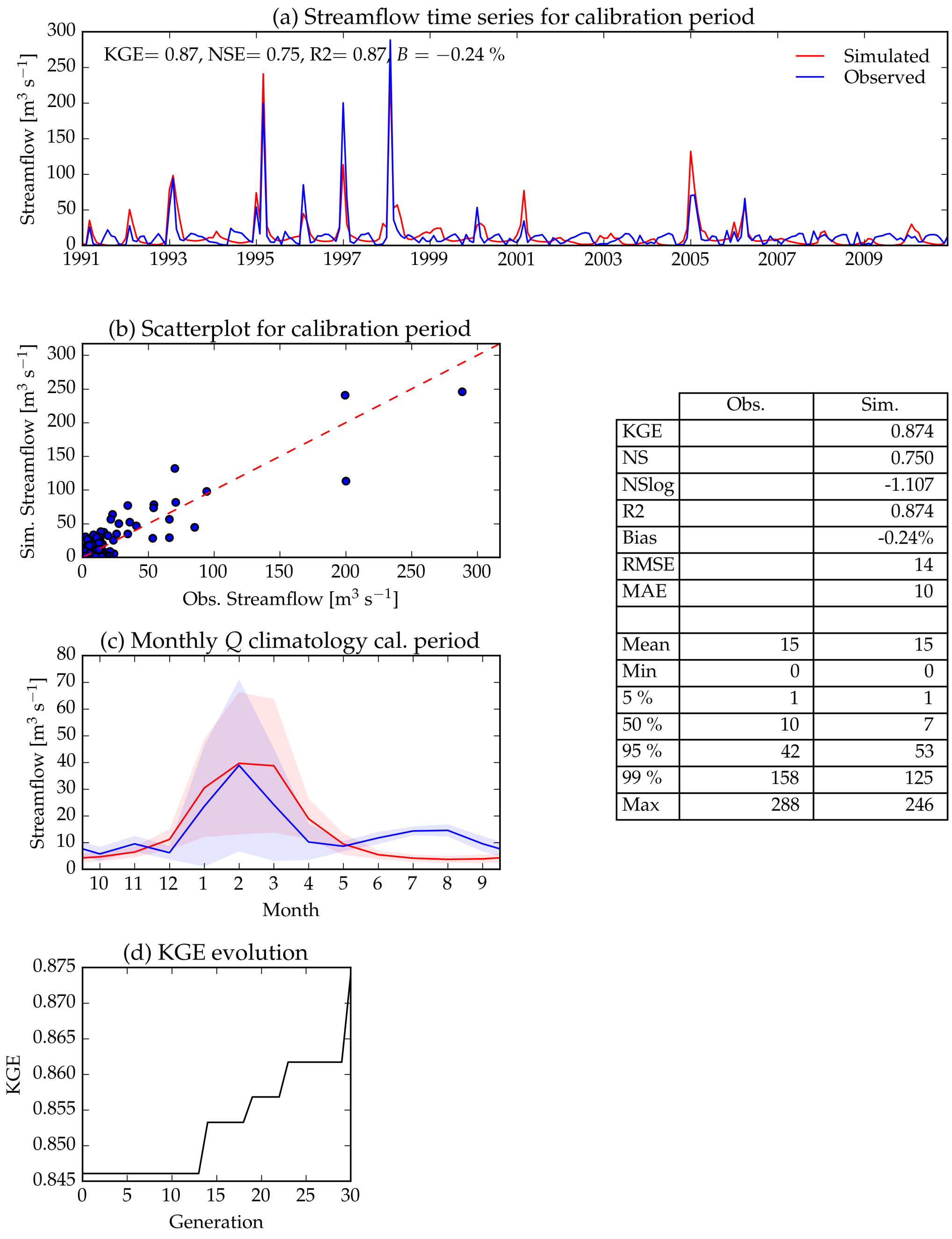

	Obs.	Sim.
KGE		0.874
NS		0.750
NSlog		-1.107
R2		0.874
Bias		-0.24%
RMSE		14
MAE		10
Mean	15	15
Min	0	0
5 %	1	1
50 %	10	7
95 %	42	53
99 %	158	125
Max	288	246

Supplementary Figure 4. Same as Supplementary Figure 2, but for USGS gauge 11150500.

Station: USGS 11150500

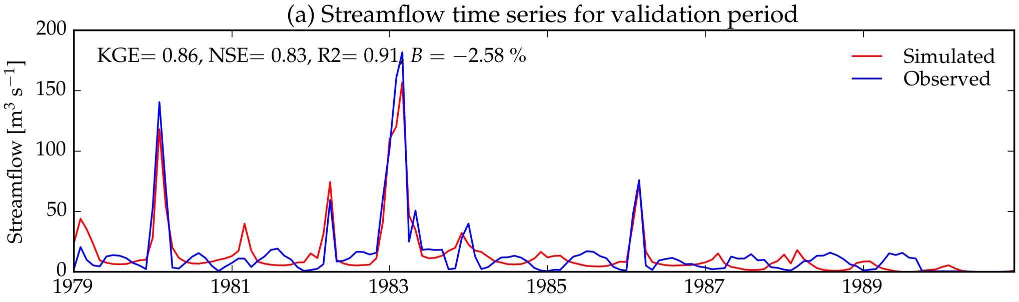

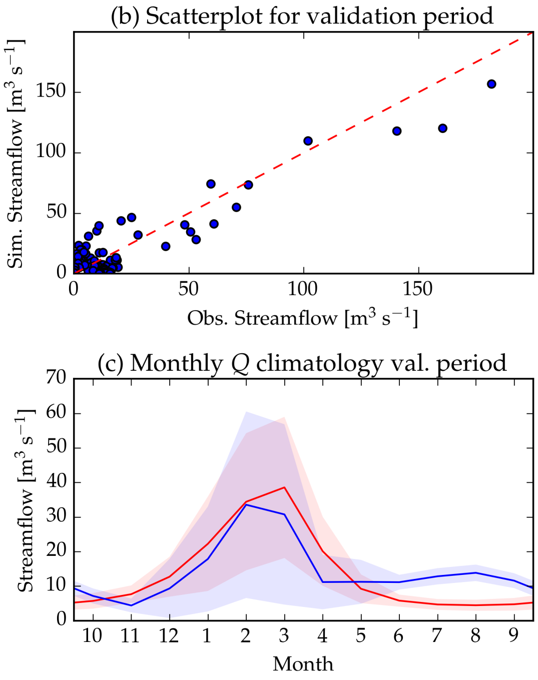

	Obs.	Sim.
KGE		0.861
NS		0.831
NSlog		nan
R2		0.912
Bias		-2.58%
RMSE		11
MAE		8
Mean	15	14
Min	0	0
5 %	0	0
50 %	9	8
95 %	59	46
99 %	152	119
Max	182	157

Supplementary Figure 5. Same as Supplementary Figure 4, but for validation.

Station: USGS 11274000

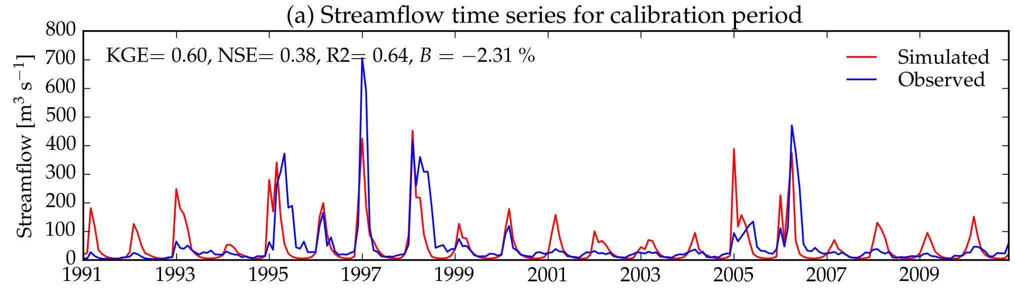

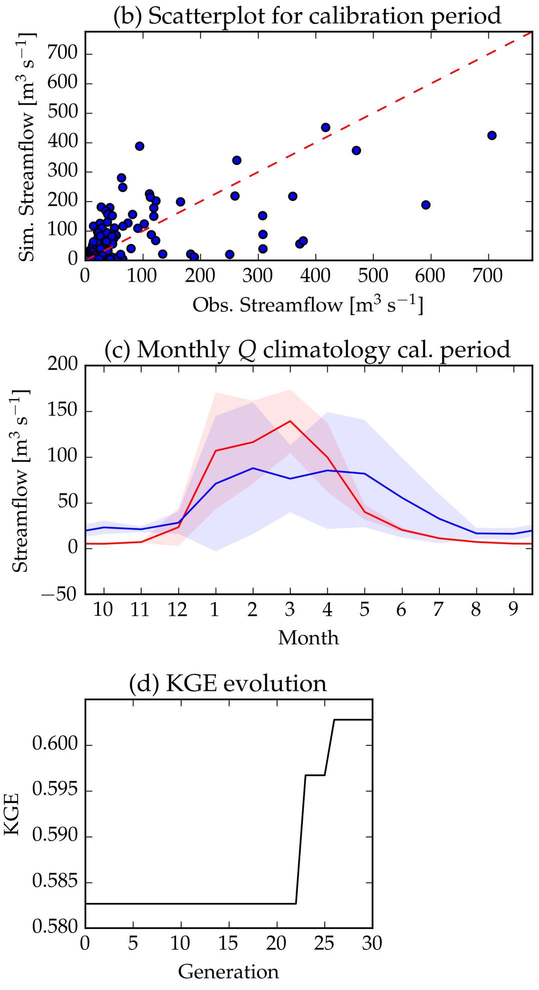

	Obs.	Sim.
KGE		0.603
NS		0.378
NSlog		0.009
R2		0.640
Bias		-2.31%
RMSE		72
MAE		37
Mean	50	49
Min	3	5
5 %	5	5
50 %	23	18
95 %	251	199
99 %	450	383
Max	706	451

Supplementary Figure 6. Same as Supplementary Figure 2, but for USGS gauge 11274000.

Station: USGS 11274000

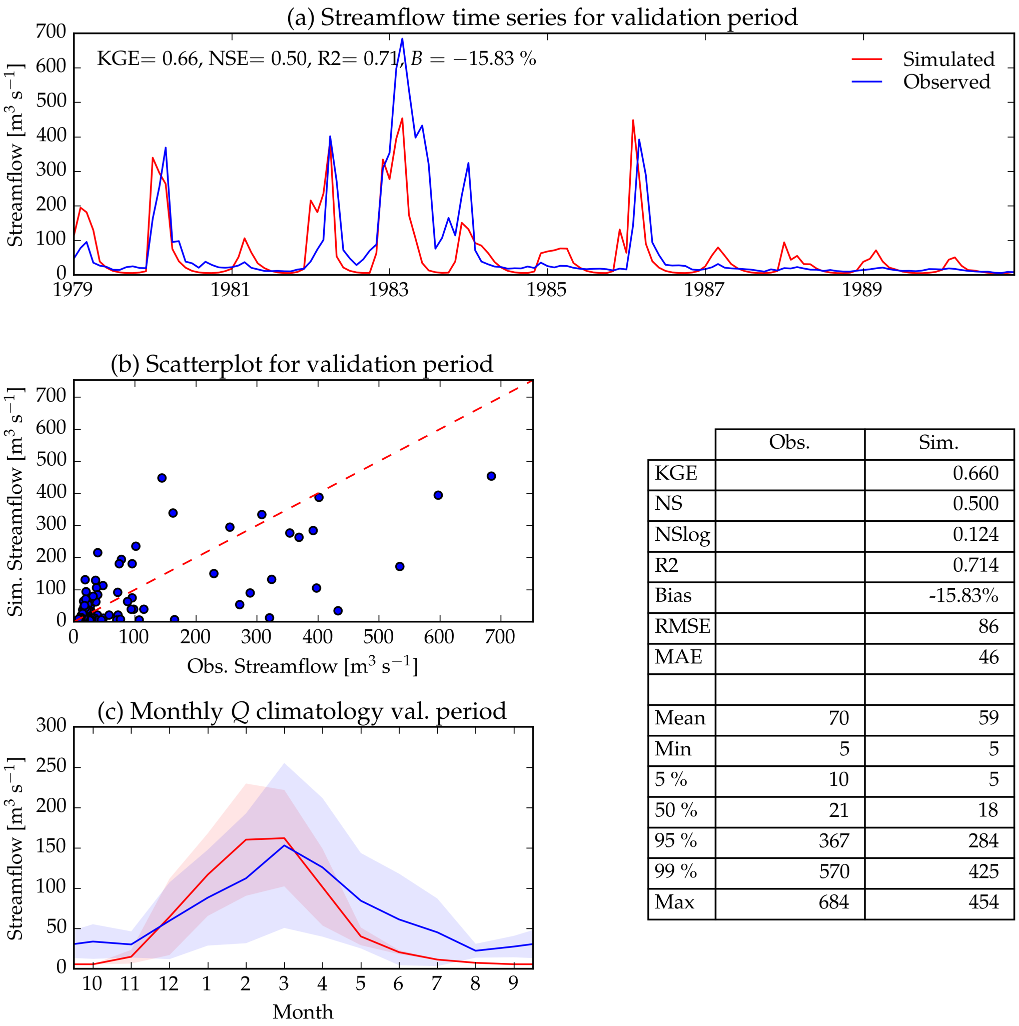

	Obs.	Sim.
KGE		0.660
NS		0.500
NSlog		0.124
R2		0.714
Bias		-15.83%
RMSE		86
MAE		46
Mean	70	59
Min	5	5
5 %	10	5
50 %	21	18
95 %	367	284
99 %	570	425
Max	684	454

Supplementary Figure 7. Same as Supplementary Figure 6, but for validation.

Station: USGS 11303500

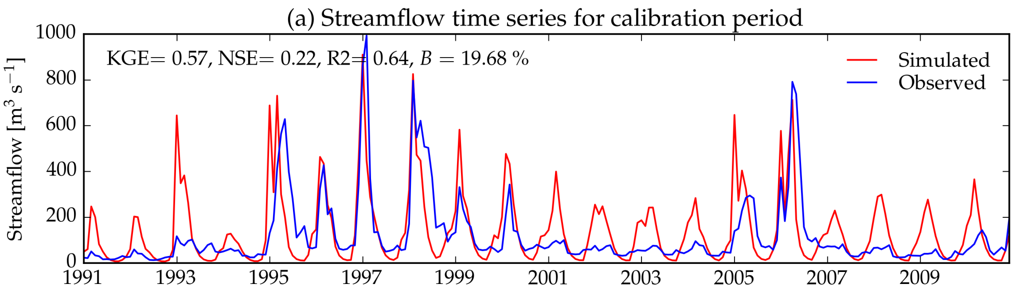

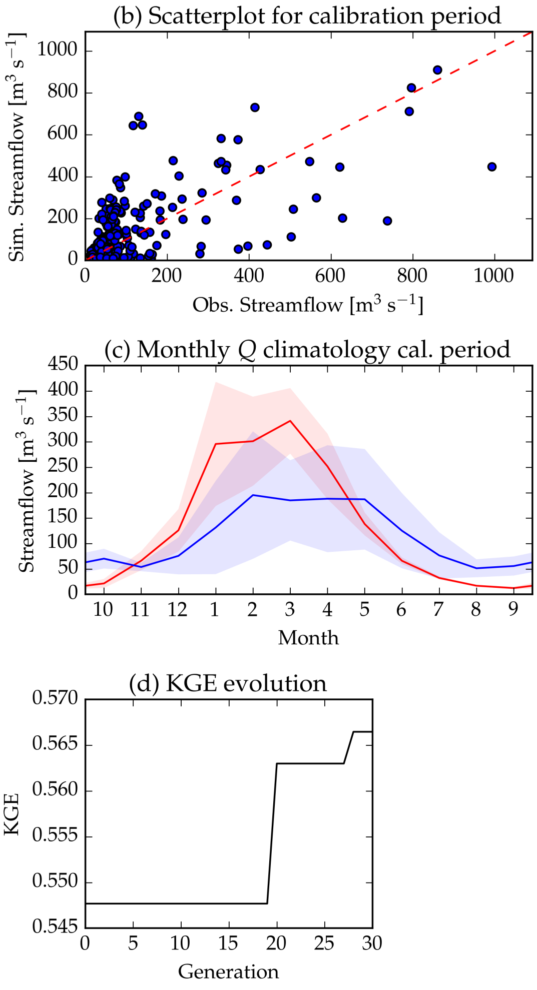

	Obs.	Sim.
KGE		0.566
NS		0.221
NSlog		-0.317
R2		0.637
Bias		19.68%
RMSE		136
MAE		87
Mean	116	139
Min	13	7
5 %	23	11
50 %	62	76
95 %	428	464
99 %	794	723
Max	993	910

Supplementary Figure 8. Same as Supplementary Figure 2, but for USGS gauge 11303500.

Station: USGS 11303500

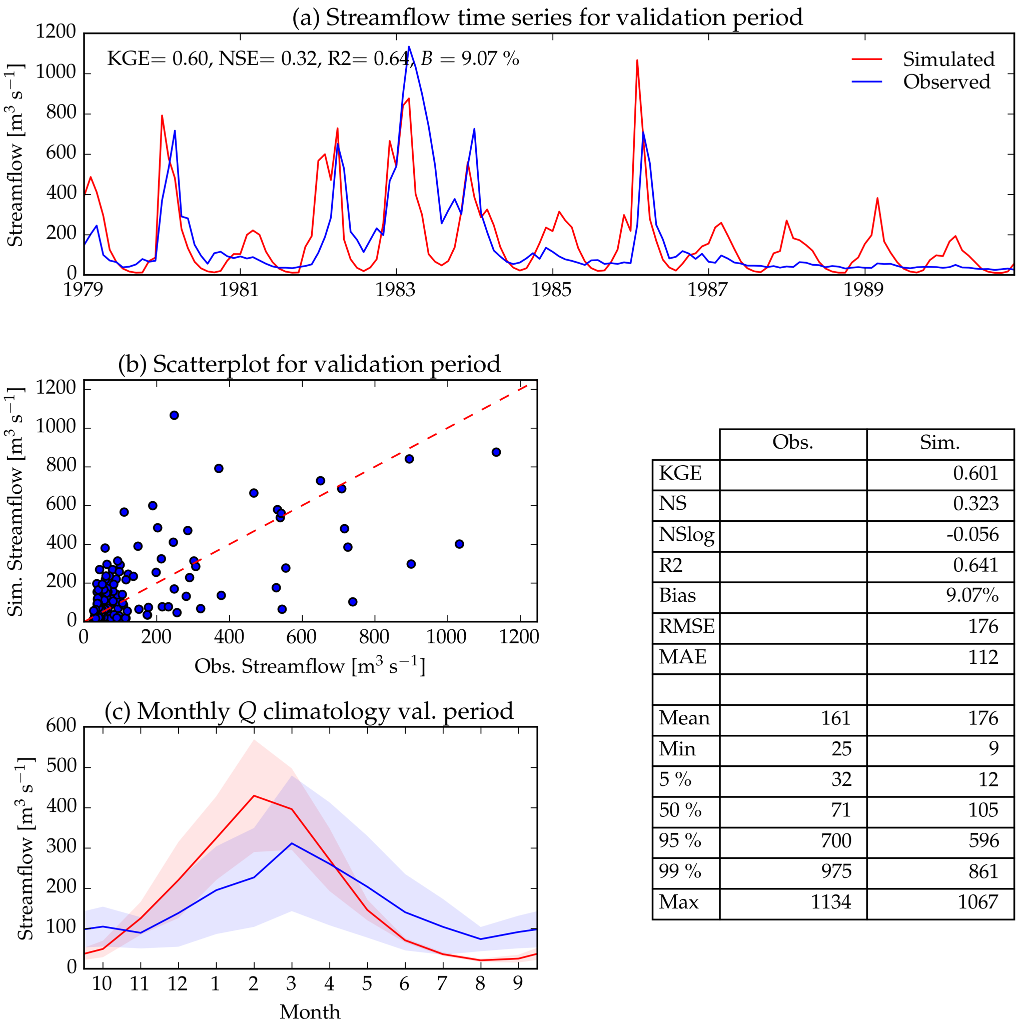

	Obs.	Sim.
KGE		0.601
NS		0.323
NSlog		-0.056
R2		0.641
Bias		9.07%
RMSE		176
MAE		112
Mean	161	176
Min	25	9
5 %	32	12
50 %	71	105
95 %	700	596
99 %	975	861
Max	1134	1067

Supplementary Figure 9. Same as Supplementary Figure 8, but for validation.

Station: USGS 11390500

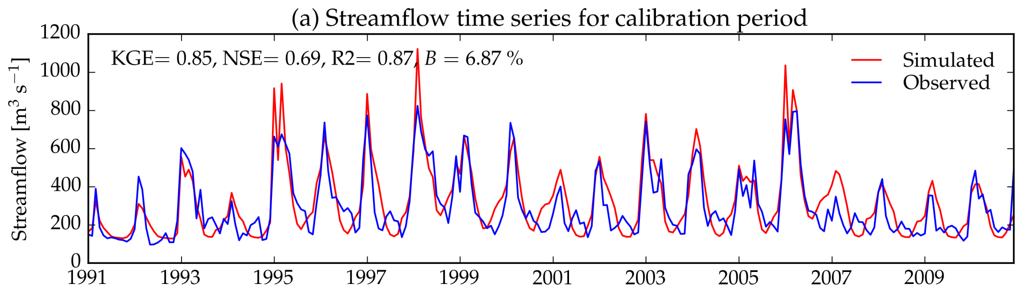

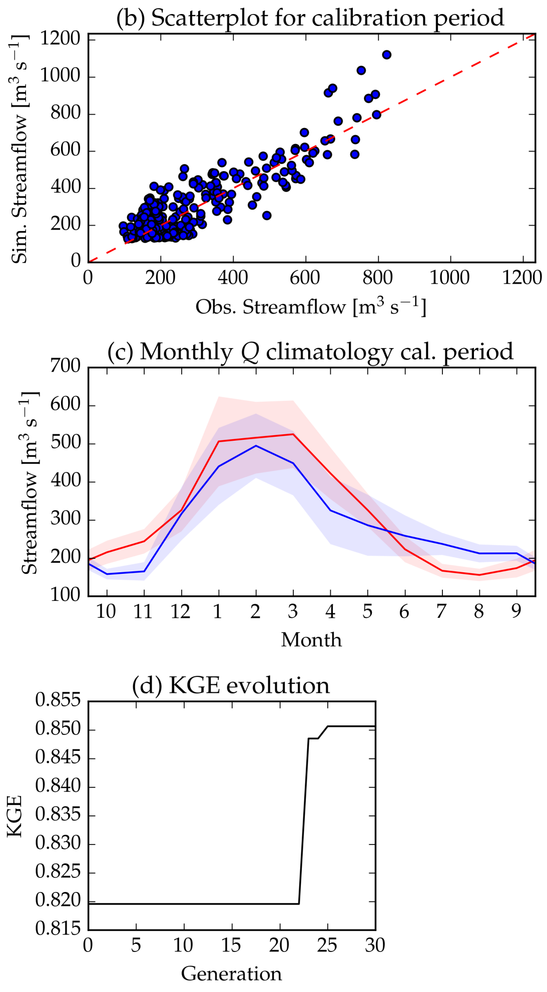

	Obs.	Sim.
KGE		0.851
NS		0.691
NSlog		0.558
R2		0.869
Bias		6.87%
RMSE		93
MAE		75
Mean	297	317
Min	96	128
5 %	126	135
50 %	234	263
95 %	660	657
99 %	785	931
Max	824	1122

Supplementary Figure 10. Same as Supplementary Figure 2, but for USGS gauge 11390500.

Station: USGS 11390500

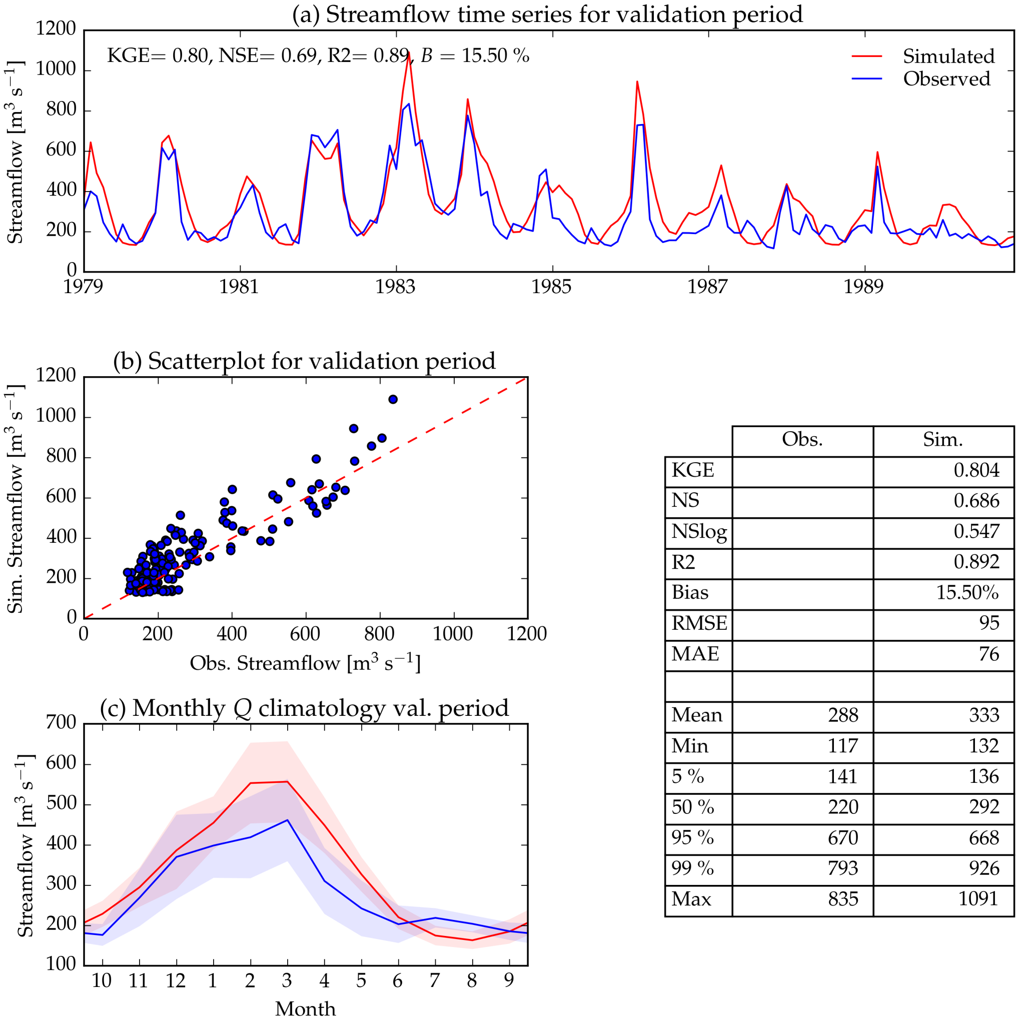

	Obs.	Sim.
KGE		0.804
NS		0.686
NSlog		0.547
R2		0.892
Bias		15.50%
RMSE		95
MAE		76
Mean	288	333
Min	117	132
5 %	141	136
50 %	220	292
95 %	670	668
99 %	793	926
Max	835	1091

Supplementary Figure 11. Same as Supplementary Figure 10, but for validation.

Station: USGS 11467000

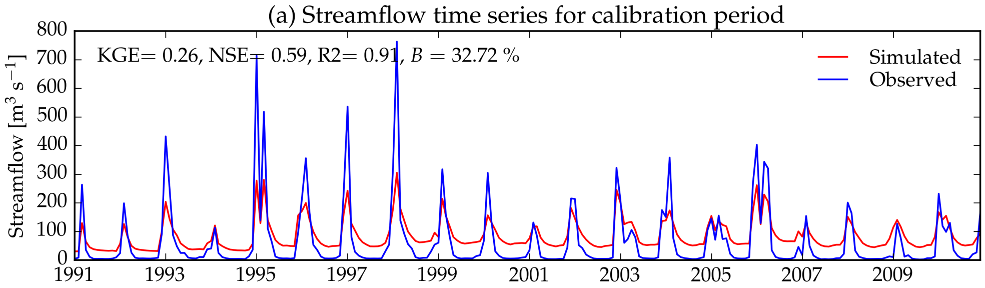

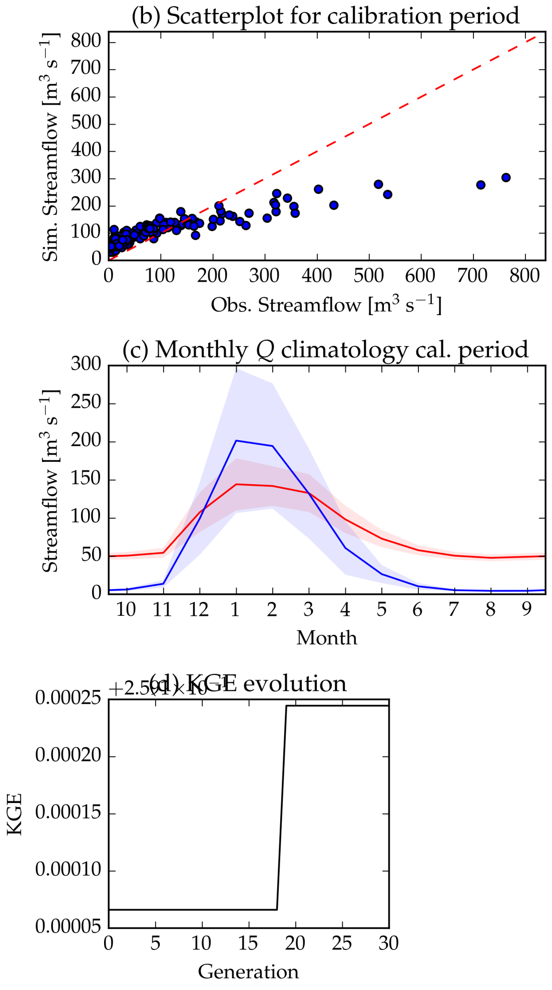

	Obs.	Sim.
KGE		0.259
NS		0.590
NSlog		-0.260
R2		0.915
Bias		32.72%
RMSE		73
MAE		53
Mean	63	84
Min	2	30
5 %	3	34
50 %	11	62
95 %	317	182
99 %	529	271
Max	763	305

Supplementary Figure 12. Same as Supplementary Figure 2, but for USGS gauge 11467000.

Station: USGS 11467000

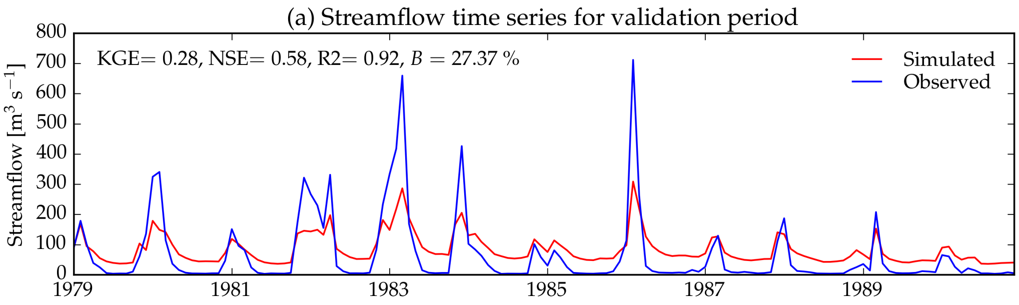

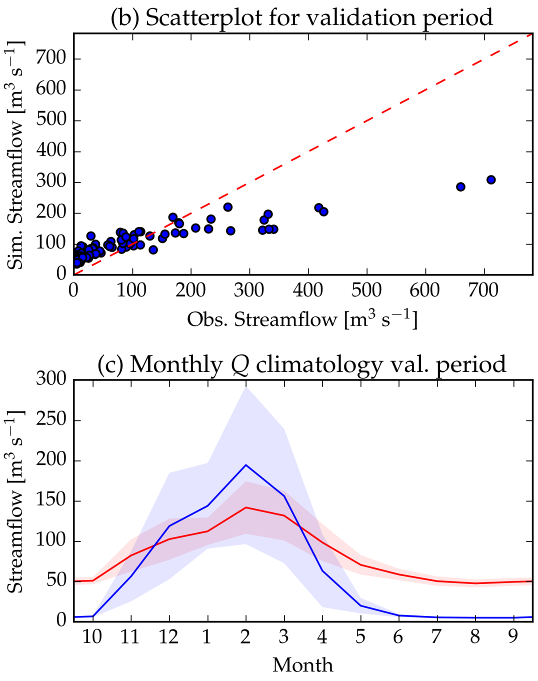

	Obs.	Sim.
KGE		0.276
NS		0.578
NSlog		-0.232
R2		0.919
Bias		27.37%
RMSE		75
MAE		54
Mean	65	83
Min	3	36
5 %	4	38
50 %	12	64
95 %	324	181
99 %	559	258
Max	712	309

Supplementary Figure 13. Same as Supplementary Figure 12, but for validation.

Station: USGS 11477000

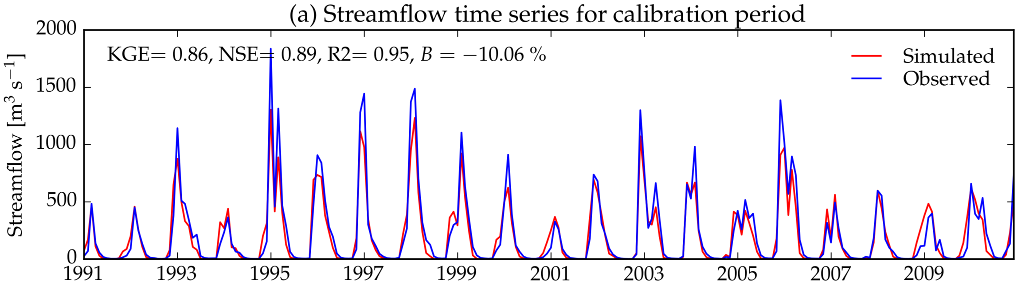

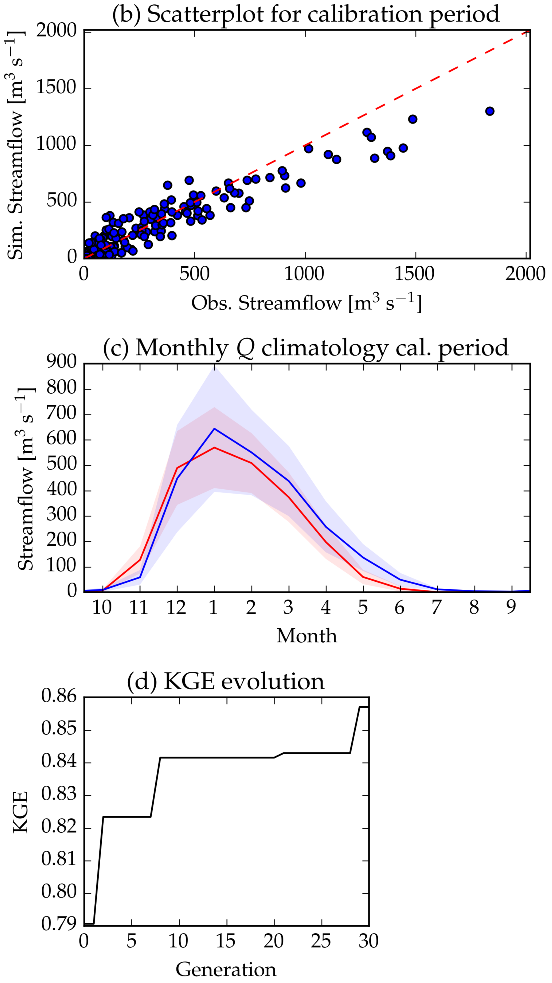

	Obs.	Sim.
KGE		0.857
NS		0.888
NSlog		-1.477
R2		0.954
Bias		-10.06%
RMSE		111
MAE		64
Mean	218	196
Min	1	0
5 %	2	0
50 %	64	55
95 %	915	736
99 %	1421	1099
Max	1836	1303

Supplementary Figure 14. Same as Supplementary Figure 2, but for USGS gauge 11477000.

Station: USGS 11477000

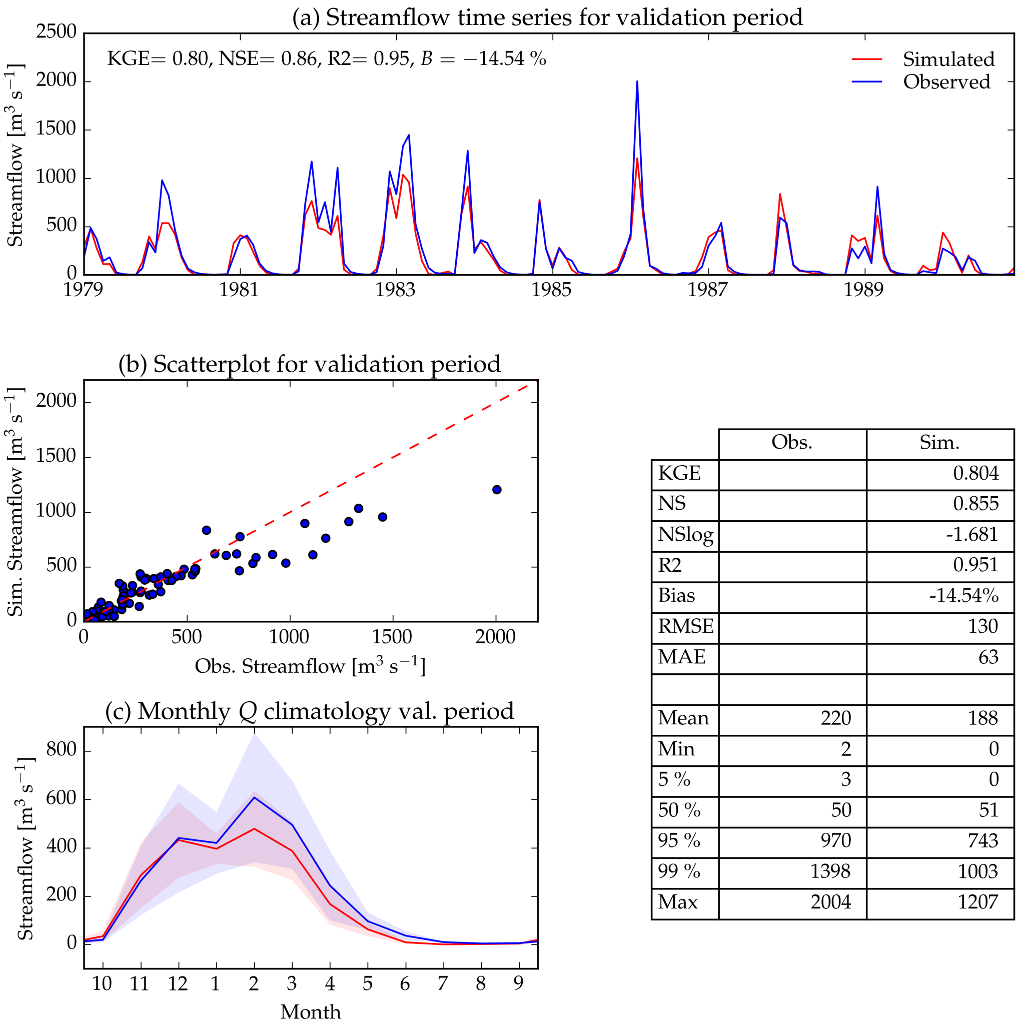

	Obs.	Sim.
KGE		0.804
NS		0.855
NSlog		-1.681
R2		0.951
Bias		-14.54%
RMSE		130
MAE		63
Mean	220	188
Min	2	0
5 %	3	0
50 %	50	51
95 %	970	743
99 %	1398	1003
Max	2004	1207

Supplementary Figure 15. Same as Supplementary Figure 14, but for validation.

River: Klamath, Station: USGS 11523000 - Orleans, CA

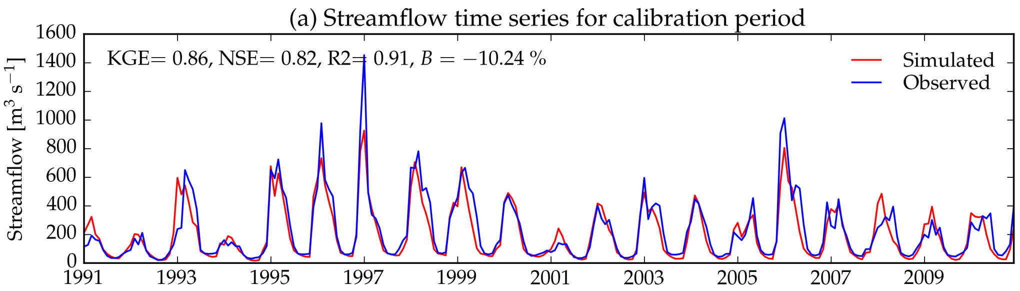

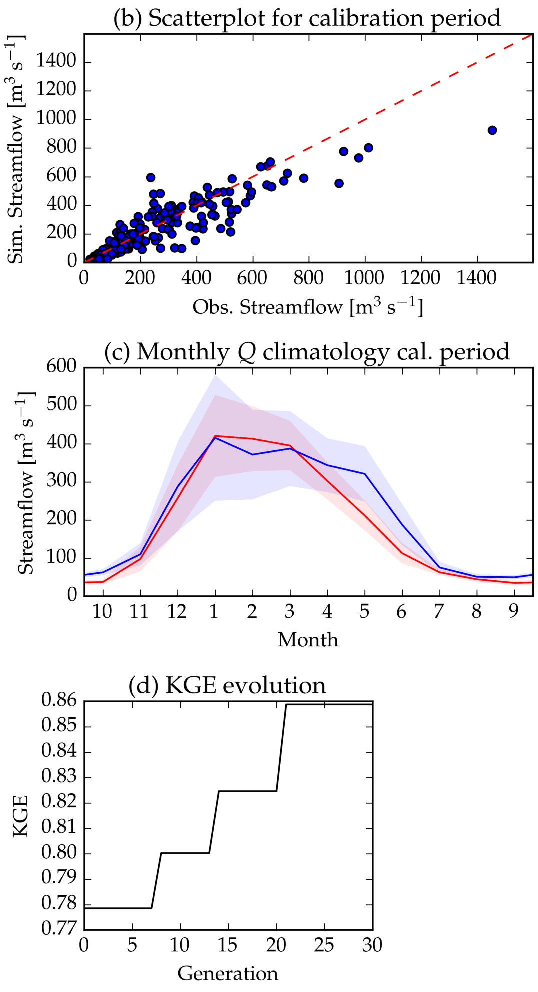

	Obs.	Sim.
KGE		0.859
NS		0.820
NSlog		0.826
R2		0.913
Bias		-10.24%
RMSE		91
MAE		57
Mean	222	200
Min	20	17
5 %	38	26
50 %	132	126
95 %	649	555
99 %	956	761
Max	1452	925

Supplementary Figure 16. Same as Supplementary Figure 2, but for USGS gauge 11523000.

River: Klamath, Station: USGS 11523000 - Orleans, CA

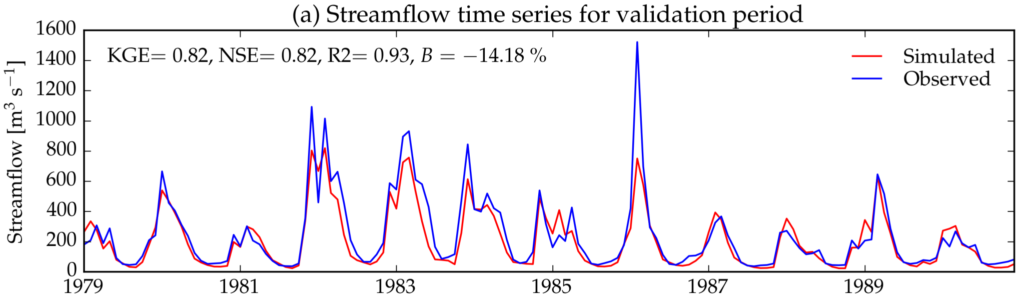

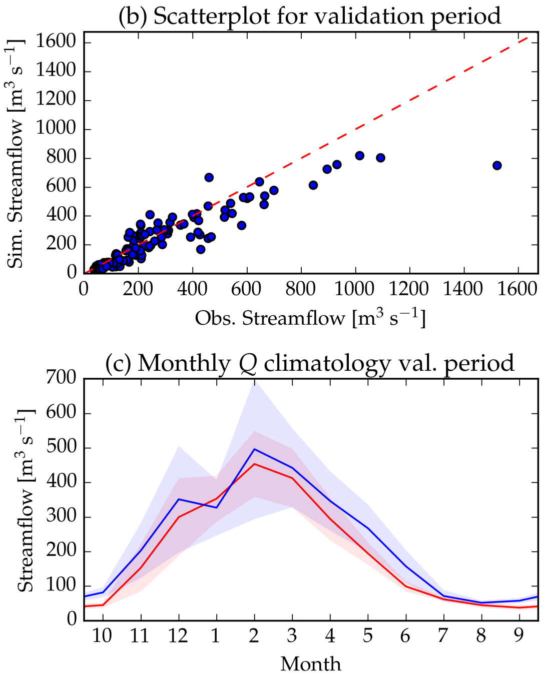

	Obs.	Sim.
KGE		0.821
NS		0.818
NSlog		0.837
R2		0.925
Bias		-14.18%
RMSE		103
MAE		58
Mean	238	204
Min	37	23
5 %	45	30
50 %	164	135
95 %	665	608
99 %	1059	783
Max	1522	819

Supplementary Figure 17. Same as Supplementary Figure 16, but for validation.

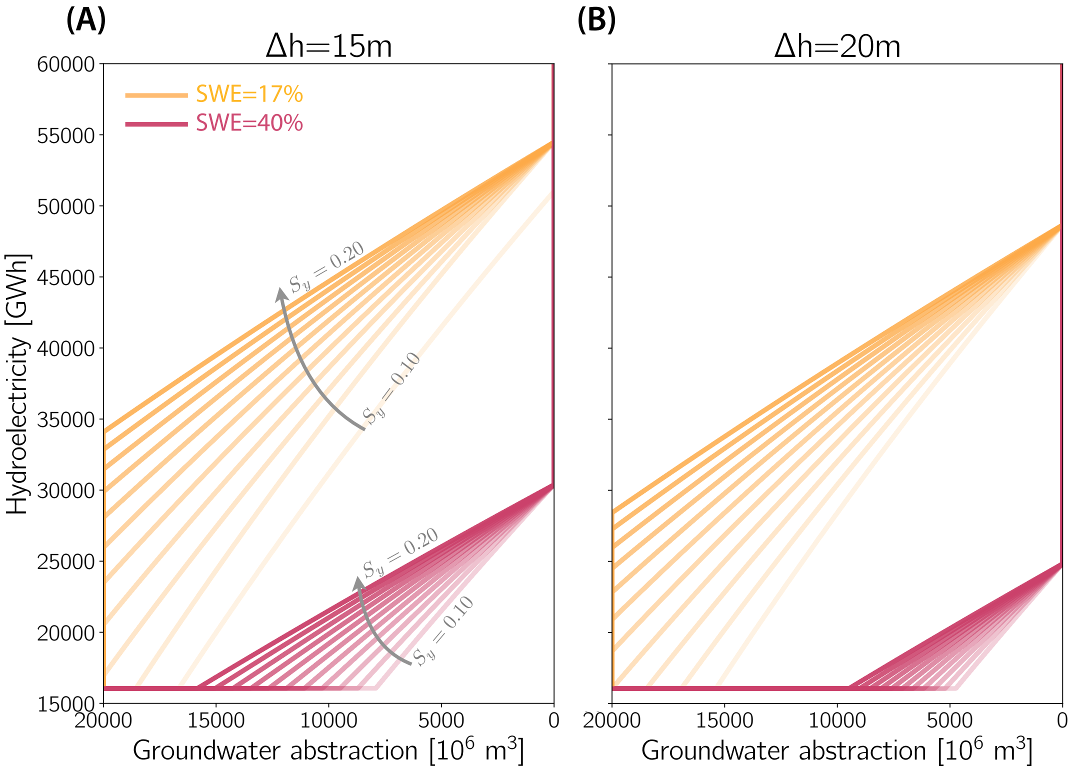

Supplementary Figure 18. Sensitivity of expansion paths (EPs) to specific yield s_y under current (yellow lines, 17%) and future (red lines, 40%) penetration of SWE with different groundwater pumping lift (Δh): (a) $\Delta h = 15m$, (b) $\Delta h = 20m$. Higher values of s_y shift the EP to the left as groundwater pumping costs are reduced, which leads to higher amount of groundwater abstraction. Here s_y ranges from 0.10 to 0.20 based on the C2VSim model[2].

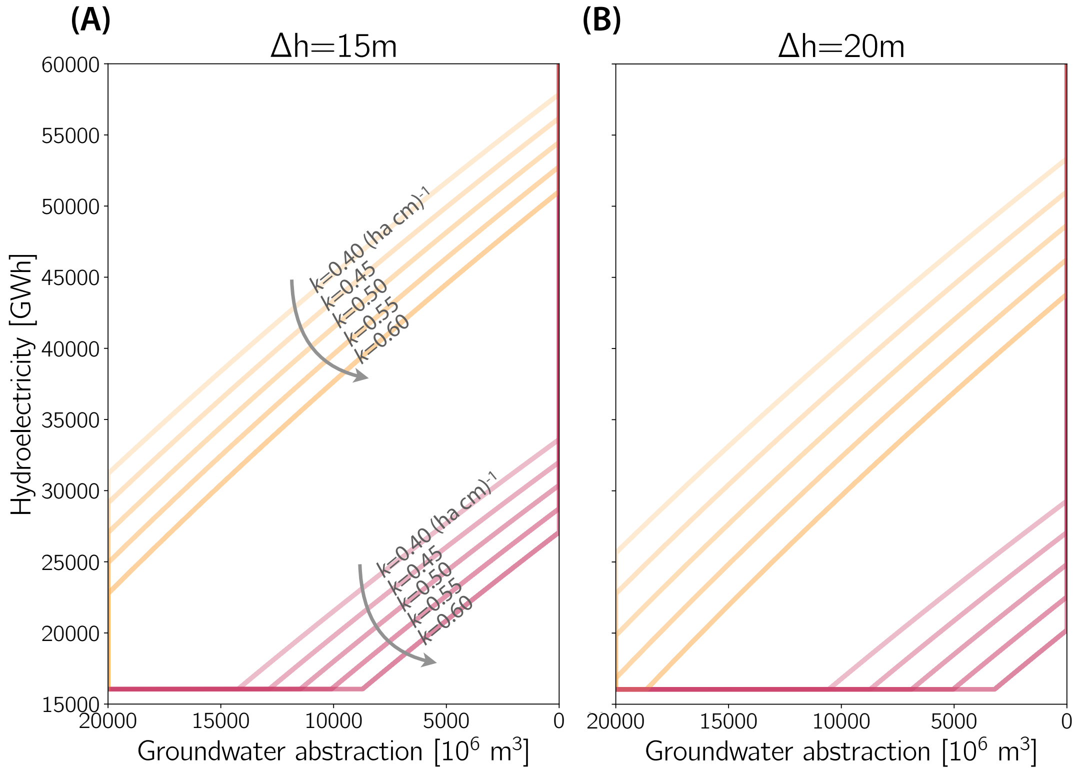

Supplementary Figure 19. Same as Supplementary Figure 18, but showing the sensitivity of EP to energy cost parameter k (related to equipment use) ranging from 0.40 to 0.60 (ha cm)$^{-1}$. With higher k values, EP moves rightward, because higher equipment use makes groundwater more expensive and therefore leads to reduced groundwater abstraction.

Supplementary Note 1. Economic revenues and costs

Economic profits from hydropower (B^{Hydro} [\$]) can be calculated as:

$$B^{\text{Hydro}}(s_w^{\text{Hydro}}) = \min\{Q_e^{\text{Total}} \times (1 - \text{pct}^{\text{SWE}}) - Q_e^{\text{Base}}, Q_e^{\text{Hydro}}\} \times p_s^{\text{Hydro}}$$

Estimation of B^{Hydro} requires to carefully price s_w^{Hydro}. Here we do not use the levelized cost of electricity (LCOE) for hydropower, instead we attempt to calculate its shadow price (p_s^{Hydro}, [\$/GWh]) given the fact that when water is limited (e.g., during drought), we must make a choice between water used for agriculture or water used for hydropower. At the annual time scale, the reduction of hydropower should be made up by natural gas according to the dispatch curve. Therefore, p_s^{Hydro} should depend on the fluctuation of natural gas price (p^{Gas}, [\$/GWh]) and can be calculated as:

$$p_s^{\text{Hydro}} = p^{\text{Gas}} \times [1 - f(\frac{Q_e^{\text{SWE}}}{Q_e^{\text{Total}}}) \times g(\frac{Q_e^{\text{Hydro}}}{Q_e^{\text{Total}}})]$$

where $f(\frac{Q_e^{\text{SWE}}}{Q_e^{\text{Total}}}) = e^{-\frac{\alpha_1 \times Q_e^{\text{Total}} \times (1-\text{pct}^{\text{SWE}})}{Q_e^{\text{Total}}}}$ is the downward adjustment of p^{Gas} due to the penetration of SWE (in other words, SWE has price advantage compared to hydropower). $g(\frac{Q_e^{\text{Hydro}}}{Q_e^{\text{Total}}}) = e^{\frac{\alpha_2 \times Q_e^{\text{Hydro}}}{Q_e^{\text{Total}}}}$ is the downward adjustment of p^{Gas} due to the reduction of hydropower (in other words, hydropower has price advantage compared to natural gas). Q_e^{Total} [GWh] is the total electricity demand for California, pct^{SWE} [-] is the percentage mix of SWE in the energy portfolios (i.e., 17% for 2016 and ∼40% by 2030), Q_e^{Base} [GWh] is the base load (e.g., nuclear), Q_e^{Hydro} [GWh] is the hydroelectricity generation and is estimated using a linear function of annual averaged streamflow[3–5]. α_1(=1) and α_2(=1.1) are scaling factors of price elasticity. It should be noted that our analysis is conducted at the annual time scale. Therefore, it is not necessary to consider the intermittency of hydropower, solar and wind energy, which in reality can be problematic to be dispatched. Nonetheless, the objective here is to examine the cumulative effects of the penetration of solar and wind on the water-food-energy nexus in a long term, which we argue that the intermittency will not influence the final results.

Groundwater pumping costs (C^{Pump} [\$]) are estimated using the following form based on[6]:

$$C^{\text{Pump}}(g_w) = (k + c\sigma)g_w + g_w c\Delta h + \frac{c}{As_y}\frac{g_w^2}{2}$$

where k [$\$/m^3$] is the average cost per unit of groundwater withdrawal related to equipment use, c [$\$/m/m^3$] is pumping costs per unit lift of extracted groundwater, σ [m] is drawdown, Δh [m] is pumping lift, A [m^2] is the aquifer area and s_y is the specific yield of the aquifer. Due to lack of reliable datasets, parameter values are either taken from the literature or derived from model input parameters. For instance, based on[7], the average groundwater pumping cost, c, in California is \$0.20 per acre-feet per feet of lift in year 2000 dollars. We adjust this value for inflation to calculate the unit pumping cost. As for s_y, we calculate the area-weighted value based on the s_y map provided by the C2VSim hydrological model[2]. Given the high spatial variability of s_y over California[2], sensitivity tests are performed to examine the robustness of optimal expansion paths (EPs) to s_y (see Supplementary Figure 18). As there are no reliable and consistent estimates for k across California, we took the value from Table A1 in[6], but with a ±20% variation of the original value to account for parameter uncertainty (see Supplementary Figure 19). Sensitivity tests (Supplementary Figure 18 and 19) show that the EPs are sensitive to s_y and k. However, our general conclusions are still robust to such sensitivity, as there is a clear separation between future and current EPs with future EPs always being to the right side of current EPs. In other words, regardless of s_y, higher penetration of SWE always reduces the groundwater abstraction and therefore increases groundwater sustainability. Although our general conclusion is not influenced by choosing different parameters, these sensitivity tests highlight the need to collect reliable and consistent datasets to appropriately parameterize s_y and k if the trade-off framework is to be applied in different regions.

Crop damages (D^{Crop} [\$]) can be estimated as:

$$D^{\text{Crop}}(s_w^{\text{Crop}}, g_w) = B^{\text{Crop}} \times (1 - \frac{s_w^{\text{Crop}} + g_w}{\text{IWR}})$$

where B^{Crop} [\$] is the averaged revenue of field crops based on the estimation from[8].

Supplementary Note 2. Physical model description (meteorological forcings and key parameters)

Precipitation, relative humidity, long- and short-wave downward surface radiation fluxes, maximum, minimum and average 2 m temperature, 10 m wind speed and surface pressure are used as the inputs to drive CWatM. We use elevation data from the Shuttle Radar Topography Mission - SRTM[9] for latitudes below and equal 60° North and HYDRO1k (US Geological Survey Center for Earth Resources Observation and Science; https://lta.cr.usgs.gov/HYDRO1K) for regions above 60° North. Soil information is obtained from ISRIC SoilGrids1km database[10]. Data used for reservoir operation (water supply, flood control, hydropower generation and others) are obtained from the HydroLakes database[11,12]. Crop-specific calendars and growing season lengths are derived from the MIRCA2000 data set[13]. For each crop, the crop coefficient at each development stage and the corresponding maximum crop rooting depth are obtained from the Global Crop Water Model (Siebert and Doll, 2010). The original 26 crop types in MIRCA2000 are reclassified into two crop classes, paddy rice and nonpaddy crop. Parameters for the nonpaddy crop are aggregated by weighting the area of each crop type. Time series of historical (1979-2010) irrigation area are spatially downscaled to 0.5° resolution from the country-level statistics (available at FAOSTAT, http://www.fao.org/faostat/en/#data/RL) based on the distribution of the gridded irrigation area in the MIRCA2000 data set (Portmann et al., 2010). Historical (1979-2010) water demand data in the household and livestock sectors are estimated and corrected based on the FAO statistics, taking into account the population growth, socioeconomic and technological development. Industry water demand data for 2000 (as baseline) are firstly obtained from[14–16], which are then reconstructed by scaling with the time series of water use intensities based on the algorithm developed by[17]. Urban water demand is linked to population densities, which are connected to the urban water supply network. In our coupled hydro-economic optimization framework, we assume that pixel-level urban water demands are satisfied first. The remaining available surface water at the pixel level is then aggregated to the whole California. This aggregated total surface water availability is used as the constraint in the optimization model. Details on how to estimate these water demand data can be found in[18].

Supplementary Note 3. Calculation of IWR for paddy and nonpaddy crops

In CWatM, irrigation water for nonpaddy crop fields, $\mathrm{IWR_{nonpaddy}}$ [m], is estimated using the following equation[19]:

$$\mathrm{IWR_{nonpaddy}} = \begin{cases} \mathrm{TAW} - \mathrm{RAW} & \mathrm{RAW} < p \times \mathrm{TAW} \\ 0 & \mathrm{RAW} > p \times \mathrm{TAW} \end{cases}$$

where TAW (total available water, [m]) is the total soil moisture available to irrigate crops in the soil column and can be calculated as:

$$\begin{aligned} \mathrm{TAW} = & \left\{ (\theta_{\mathrm{E,FC}_{S_1}} - \theta_{\mathrm{E,wp}_{S_1}}) \times (\theta_{\mathrm{sat}_{S_1}} - \theta_{\mathrm{res}_{S_1}}) \times \min(\mathrm{SC}_{S_1}, Z_r) \right\} \\ & + \left\{ (\theta_{\mathrm{E,FC}_{S_2}} - \theta_{\mathrm{E,wp}_{S_2}}) \times (\theta_{\mathrm{sat}_{S_2}} - \theta_{\mathrm{res}_{S_2}}) \times \min(\mathrm{SC}_{S_2}, \max(0, Z_r - \mathrm{SC}_{S_1})) \right\} \end{aligned}$$

and RAW (readily available water, [m]) is the actual soil moisture that are available in the root zone:

$$\begin{aligned} \mathrm{RAW} = & \left\{ (\theta_{\mathrm{E}_{S_1}} - \theta_{\mathrm{E,wp}_{S_1}}) \times (\theta_{\mathrm{sat}_{S_1}} - \theta_{\mathrm{res}_{S_1}}) \times \min(\mathrm{SC}_{S_1}, Z_r) \right\} \\ & + \left\{ (\theta_{\mathrm{E}_{S_2}} - \theta_{\mathrm{E,wp}_{S_2}}) \times (\theta_{\mathrm{sat}_{S_2}} - \theta_{\mathrm{res}_{S_2}}) \times \min(\mathrm{SC}_{S_2}, \max(0, Z_r - \mathrm{SC}_{S_1})) \right\} \end{aligned}$$

where

$\theta_{\mathrm{E,FC}}$	:	Effective degree of saturation at field capacity	[–]
$\theta_{\mathrm{E,wp}}$	:	Effective degree of saturation at wilting point	[–]
θ_{sat}	:	Saturated (volumetric) water content	$[\mathrm{m^3/m^3}]$
θ_{res}	:	Residual (volumetric) water content	$[\mathrm{m^3/m^3}]$
SC	:	Storage capacity of the soil layer	[m]
Z_r	:	Rooting depth	[m]
$\mathrm{S_1}$	:	First soil layer	[/]
$\mathrm{S_2}$	:	Second soil layer	[/]

p [–] is the fraction of easily available soil water which crops can extract from the root zone before suffering moisture stress. It can be calculated using the following empirical formula:

$$p = \frac{1}{\alpha_p + \beta_p \times \mathrm{ET_0}} - 0.1 \times (5 - \mathrm{No_{cg}})$$

where

α_p	:	Regression constant [=0.76][20]	[–]
β_p	:	Regression constant [=1.5][20]	[–]
$\mathrm{ET_0}$	:	Potential evapotranspiration rate	[cm/day]
$\mathrm{No_{cg}}$	:	Crop group number [=1 to 5][21]	[–]

For paddy crop, we estimate irrigation water requirement ($\mathrm{IWR_{paddy}}$ [m]) at time t and associated surface water balance using the following equations:

$$\begin{aligned} \mathrm{IWR_{paddy,t}} &= \max(0, S_{\max} - (S_{0,t-1} + P_{\mathrm{net},t})) \\ S_{0,t} &= S_{0,t-1} + P_{\mathrm{net},t} + \mathrm{IWR_{paddy,t}} - q_{i,S_0 \to S_1,t} - \mathrm{EW}_{S_0,t} \end{aligned}$$

where

$S_{\max}$	:	Maximum surface water depth over the paddy fields (= 0.05)	[m]
$S_{0,t}$	:	Surface water layer over the paddy fields at time t	[m]
$P_{\mathrm{net},t}$	:	Net liquid precipitation	[m]
$q_{i,S_0 \to S_1,t}$	:	Infiltration from surface water layer (S_0) to first soil layer (S_1) at time t	[m]
$\mathrm{EW}_{S_0,t}$	:	Open water evaporation from the surface water layer (S_0)	[m]

Supplementary Note 4. Pseudocode for the trade-off analysis

To find the optimal water allocation (solid point in Figure 1) given the water constraint, we first calculate the water allocation efficiency (WAE) for hydropower ($\text{WAE}^{\text{Hydro}}$) and crop ($\text{WAE}^{\text{Crop}}$) using the following equations:

$$\text{WAE}^{\text{Hydro}} = \frac{R(s_w^{\text{Hydro}} + \delta s_w^{\text{Hydro}}, s_w^{\text{Crop}}, g_w) - R(s_w^{\text{Hydro}}, s_w^{\text{Crop}}, g_w)}{\delta s_w}$$

$$\text{WAE}^{\text{Crop}} = \frac{R(s_w^{\text{Hydro}}, s_w^{\text{Crop}} + \delta s_w^{\text{Crop}}, g_w) - R(s_w^{\text{Hydro}}, s_w^{\text{Crop}}, g_w)}{\delta s_w}$$

Water allocation decisions will be made based on the relative value of $\text{WAE}^{\text{Hydro}}$ and WAE^{Crop}. Assume that at the initial stage, WAE^{Crop} is larger than $\text{WAE}^{\text{Hydro}}$, then within a certain range of water availability, water will only be allocated for irrigation purpose. However, as the water availability increases, WAE^{Crop} decreases. When WAE^{Crop} reaches to the same value as $\text{WAE}^{\text{Hydro}}$, water will start to be allocated for both crop irrigation and hydropower. When we implement this algorithm, we do not have to explicitly calculate the value of $\text{WAE}^{\text{Hydro}}$ and WAE^{Crop} as we may face numerical stability problems if δs_w is very small. Instead, we just need to compare $R(s_w^{\text{Hydro}} + \delta s_w^{\text{Hydro}}, s_w^{\text{Crop}}, g_w)$ and $R(s_w^{\text{Hydro}}, s_w^{\text{Crop}} + \delta s_w^{\text{Crop}}, g_w)$ as we can get rid of $R(s_w^{\text{Hydro}}, s_w^{\text{Crop}}, g_w)$ and δs_w if we let $\text{WAE}^{\text{Hydro}}$ = WAE^{Crop}. In the following, we consider two cases to solve the nonlinear optimization problem with and without consideration of groundwater cap (see algorithms in Supplementary Note 5 and 6).

Supplementary Note 5

Algorithm 1 Using implicit gradient descent approach to find the optimal point **(No groundwater cap)**

Input:

s_w: surface water availability
δs_w: step size
$\varepsilon_0 (< 1)$: accuracy limit
IWR: irrigation water requirement

Output:

s_w^{Hydro}: surface water for hydropower
s_w^{Crop}: surface water for crop irrigation
g_w: groundwater withdrawal

function FINDOPTIMALPOINT(s_w, δw, ε_0, IWR)
    $s_{w,0}^{\text{Hydro}} = s_{w,0}^{\text{Crop}} = 0,\ s_{w,0} = s_w - s_{w,0}^{\text{Hydro}} - s_{w,0}^{\text{Crop}}, g_{w,0} = \text{IWR}$ */* Initialize water allocation */*
    while $s_{w,0} > 0$ **do**
        if $s_{w,0}^{\text{Crop}} > \text{IWR}$ **then**   */* If water allocation to crop is over the total demand */*
            $\varepsilon = 0$ */* Set error to be 0 */*
            $s_{w,0}^{\text{Hydro}} = s_{w,0}^{\text{Hydro}} + \delta s_w$ */* Allocate water to hydropower */*
        else
            $k = 0, \gamma_0 = 1, \varepsilon = 1$ */* Initialize the iteration number k and allocation parameter γ */*
            while $\varepsilon > \varepsilon_0$ **do**   */* While the result is not precise, keep this loop */*
                $s_{w,k+1}^{\text{Hydro}} = s_{w,k}^{\text{Hydro}} + \gamma_k \delta s_w$ */* Allocate water to hydropower */*
                $s_{w,k+1}^{\text{Crop}} = s_{w,k}^{\text{Crop}} + (1 - \gamma_k)\delta s_w$ */* Allocate water to crop */*
                $g_{w,k+1} = \text{IWR} - s_{w,k+1}^{\text{Crop}}$ */* Calculate groundwater withdrawal */*
                $\text{WAE}_k^{\text{Hydro}} = R(s_{w,k+1}^{\text{Hydro}}, s_{w,k}^{\text{Crop}}, g_{w,k})$ */* Calculate allocation efficiency for hydropower */*
                $\text{WAE}_k^{\text{Crop}} = R(s_{w,k}^{\text{Hydro}}, s_{w,k+1}^{\text{Crop}}, g_{w,k+1})$ */* Calculate allocation efficiency for crop */*
                if $\text{WAE}_k^{\text{Hydro}} < \text{WAE}_k^{\text{Crop}}$ **then**   */* If the allocation efficiency differs */*
                    $\gamma_{k+1} = \gamma_k - 2^{-k-1}$ */* Adjust the allocation */*
                else
                    $\gamma_{k+1} = \gamma_k + 2^{-k-1}$
                    if $\gamma_{k+1} > 1$ **then**
                        break
                    end if
                end if
                $\varepsilon = |\text{WAE}_k^{\text{Hydro}} - \text{WAE}_k^{\text{Crop}}|$ */* Calculate the error */*
                $k = k + 1$ */* Increase the iteration number */*
            end while
            $s_{w,0} = s_{w,0} - \delta s_w$ */* Change the remained surface water */*
            $s_{w,0}^{\text{Hydro}} = s_{w,k}^{\text{Hydro}},\ s_{w,0}^{\text{Crop}} = s_{w,k}^{\text{Crop}}$ */* Re-initialization */*
        end if
    end while
    $s_w^{\text{Hydro}} = s_{w,0}^{\text{Hydro}},\ s_w^{\text{Crop}} = s_{w,0}^{\text{Crop}},\ g_w = \text{IWR} - s_w^{\text{Crop}}$ */* Find the optimal solution */*
    return $s_w^{\text{Hydro}}, s_w^{\text{Crop}}, g_w$
end function

Supplementary Note 6

Algorithm 2 Using implicit gradient descent approach to find the optimal point **(With groundwater cap)**

Input:
s_w: surface water availability
δs_w: step size
$\varepsilon_0 (< 1)$: accuracy limit
IWR: irrigation water requirement
g_w^{Cap}: groundwater cap

Output:
s_w^{Hydro}: surface water for hydropower
s_w^{Crop}: surface water for crop irrigation
g_w: groundwater withdrawal

function FINDOPTIMALPOINTWITHCAP(s_w, δw, ε_0, IWR, g_w^{Cap})
  $s_{w,0}^{\text{Hydro}} = s_{w,0}^{\text{Crop}} = 0$, $s_{w,0} = s_w - s_{w,0}^{\text{Hydro}} - s_{w,0}^{\text{Crop}}$ — */* Initialize water allocation */*
  $[s_w^{\text{Hydro}}, s_w^{\text{Hydro}}, g_w]$ = FINDOPTIMALPOINT(s_w, δs_w, ε_0, IWR)
  if $g_w > g_w^{\text{Cap}}$ **then**
    while $s_{w,0} > 0$ **do** — */* Allocate water step by step */*
      $k = 0, \gamma_0 = 1, \varepsilon = 1$ — */* Initialize the iteration number k and allocation parameter γ */*
      while $\varepsilon > \varepsilon_0$ **do** — */* While the result is not precise, keep this loop */*
        if $s_{w,0}^{\text{Crop}} > \text{IWR}$ **then** — */* If water allocation to crop is over the total demand */*
          $\varepsilon = 0$ — */* Set error to be 0 */*
          $s_{w,0}^{\text{Hydro}} = s_{w,0}^{\text{Hydro}} + \delta s_w$ — */* Allocate water to hydropower */*
        else
          $s_{w,k+1}^{\text{Hydro}} = s_{w,k}^{\text{Hydro}} + \gamma_k \delta s_w$ — */* Allocate water to hydropower */*
          $s_{w,k+1}^{\text{Crop}} = s_{w,k}^{\text{Crop}} + (1 - \gamma_k) \delta s_w$ — */* Allocate water to crop */*
          $\text{WAE}_k^{\text{Hydro}} = R(s_{w,k+1}^{\text{Hydro}}, s_{w,k}^{\text{Crop}}, g_w^{\text{Cap}})$ — */* Calculate allocation efficiency for hydropower */*
          $\text{WAE}_k^{\text{Crop}} = R(s_{w,k}^{\text{Hydro}}, s_{w,k+1}^{\text{Crop}}, g_w^{\text{Cap}})$ — */* Calculate allocation efficiency for crop */*
          if $\text{WAE}_k^{\text{Hydro}} < \text{WAE}_k^{\text{Crop}}$ **then** — */* If the allocation efficiency differs */*
            $\gamma_{k+1} = \gamma_k - 2^{-k-1}$ — */* Adjust the allocation */*
          else
            $\gamma_{k+1} = \gamma_k + 2^{-k-1}$
            if $\gamma_{k+1} > 1$ **then**
              break
            end if
          end if
          $\varepsilon = |\text{WAE}_k^{\text{Hydro}} - \text{WAE}_k^{\text{Crop}}|$ — */* Calculate the error */*
          $k = k + 1$ — */* Increase the iteration number */*
        end if
      end while
      $s_{w,0} = s_{w,0} - \delta s_w$ — */* Change the remained surface water */*
      $s_{w,0}^{\text{Hydro}} = s_{w,k}^{\text{Hydro}}$, $s_{w,0}^{\text{Crop}} = s_{w,k}^{\text{Crop}}$ — */* Re-initialization */*
    end while
    $s_w^{\text{Hydro}} = s_{w,0}^{\text{Hydro}}$, $s_w^{\text{Crop}} = s_{w,0}^{\text{Crop}}$, $g_w = g_w^{\text{Cap}}$ — */* Find the optimal solution */*
  end if
  return $s_w^{\text{Hydro}}, s_w^{\text{Crop}}, g_w$
end function

References

1. Cooley, H., Gleick, P. & Wilkinson, R. Agricultural water conservation and efficiency potential in california. *Issue Brief* 1–7 (2014).
2. Brush, C. F., Dogrul, E. C. & Kadir, T. N. *Development and calibration of the California Central Valley Groundwater-Surface Water Simulation Model (C2VSim), version 3.02-CG* (Bay-Delta Office, California Department of Water Resources, 2013).
3. Bartos, M. D. & Chester, M. V. Impacts of climate change on electric power supply in the Western United States. *Nat. Clim. Chang.* **5**, 748–752 (2015).
4. Perrone, D. & Hornberger, G. Frontiers of the food–energy–water trilemma: Sri Lanka as a microcosm of tradeoffs. *Environ. Res. Lett.* **11**, 014005 (2016).
5. Gleick, P. Impacts of California's five-Year (2012-2016) drought on hydroelectricity generation. *Pac. Inst.* (2017).
6. Knapp, K. C., Weinberg, M., Howitt, R. & Posnikoff, J. F. Water transfers, agriculture, and groundwater management: A dynamic economic analysis. *J. environmental management* **67**, 291–301 (2003).
7. Chou, H. *Groundwater overdraft in California's Central Valley: updated CALVIN modeling using recent CVHM and C2VSIM representations* (University of California, Davis, 2012).
8. Cooley, H., Donnelly, K., Phurisamban, R. & Subramanian, M. Impacts of California's ongoing drought: Agriculture. *Pac. Institute: Oakland, CA, USA* (2015).
9. Jarvis, A., Reuter, H. I., Nelson, A., Guevara, E. *et al.* Hole-filled SRTM for the globe Version 4. *available from CGIAR-CSI SRTM 90m Database (http://srtm. csi. cgiar. org)* **15** (2008).
10. Hengl, T. *et al.* SoilGrids1km – global soil information based on automated mapping. *PLoS One* **9**, e105992 (2014).
11. Lehner, B. *et al.* High-resolution mapping of the world's reservoirs and dams for sustainable river-flow management. *Front. Ecol. Environ.* **9**, 494–502 (2011).
12. Messager, M. L., Lehner, B., Grill, G., Nedeva, I. & Schmitt, O. Estimating the volume and age of water stored in global lakes using a geo-statistical approach. *Nat. communications* **7**, 13603 (2016).
13. Portmann, F. T., Siebert, S. & Döll, P. MIRCA2000 – Global monthly irrigated and rainfed crop areas around the year 2000: A new high-resolution data set for agricultural and hydrological modeling. *Glob. Biogeochem. Cycles* **24** (2010).
14. Shiklomanov, I. A. *Comprehensive assessment of the freshwater resources of the world: Assessment of water resources and water availability in the world* (UN, 1996).
15. World Resources: A Guide to the Global Environment 1998-99, World Resources Institute, Washington DC, USA (1998).
16. Vörösmarty, C. J., Leveque, C. & Revenga, C. *Millennium Ecosystem Assessment Volume 1: Conditions and Trends, chap. 7: Freshwater ecosystems* (Island Press, Washington DC, USA, 2005).
17. Wada, Y., van Beek, L. P. H. & Bierkens, M. F. P. Modelling global water stress of the recent past: On the relative importance of trends in water demand and climate variability. *Hydrol. Earth Syst. Sci.* **15**, 3785–3808, DOI: 10.5194/hess-15-3785-2011 (2011).
18. Wada, Y., Wisser, D. & Bierkens, M. F. P. Global modeling of withdrawal, allocation and consumptive use of surface water and groundwater resources. *Earth Syst. Dyn.* **5**, 15–40, DOI: 10.5194/esd-5-15-2014 (2014).
19. Allen, R. G., Pereira, L. S., Raes, D., Smith, M. *et al.* Crop evapotranspiration-Guidelines for computing crop water requirements – FAO irrigation and drainage paper 56. *FAO, Rome* **300**, D05109 (1998).
20. van Diepen, C., Rappoldt, C., Wolf, J. & van Keulen, H. *Crop growth simulation model WOFOST. Documentation version 4.1. for World Food Studies* (Wageningen, The Netherlands, 1988).
21. Doorenbos, J. & Kassam, A. Yield response to water. *Irrigation drainage paper* **33**, 257 (1979).
